# Urodynamic Voiding Patterns in Multiple Sclerosis

**DOI:** 10.1002/nau.70348

**Published:** 2026-06-22

**Authors:** Camille Chesnel, Nicolas Turmel, Maelys Teng, Emilie Blouet, Gérard Amarenco, Claire Hentzen

**Affiliations:** ^1^ Sorbonne Université, GRC‐01 GREEN Service de Neuro‐urologie, Hôpital Pitié Salpêtrière Paris France; ^2^ Service de Médecine Physique et de Réadaptation HDJ MPR, Hôpital René Muret, APHP Sevran France

**Keywords:** detrusor sphincter dyssynergia, lower urinary tract symptoms, multiple sclerosis, neurogenic bladder

## Abstract

**Aims:**

This study aimed to describe urodynamic voiding patterns in patients with MS (PwMS) using standardized assessments, and to compare the performance of the available nomograms and indices for obstruction and bladder contractility.

**Methods:**

PwMS and lower urinary tract symptoms underwent cystometry and pressure flow studies. Fluoroscopy findings were collected when available. Bladder contractility was assessed using the following parameters: bladder voiding efficiency (BVE), bladder contractility index (BCI), Watts factor (WF) for men, Projected Isovolumetric Pressure 1 (PIP1), the Valentini−Besson−Nelson (VBN) parameter k, and the urodynamic cut‐off proposed by Gammie et al. for women. Obstruction was assessed using the bladder outlet obstruction index (BOOI) in men, and BOOIf in women. Agreement between the diagnosis of detrusor underactivity (DUA) and obstruction according to each parameter was assessed with weight kappa.

**Results:**

Ninety‐seven PwMS were included (mean age 48.4 ± 10.5, 58 [60%] women, mean EDSS 3.9 ± 1.8). Sphincter dysfunction was observed in 68 patients (70%), most frequently detrusor sphincter dyssynergia (DSD) in 32 (33%), followed by non‐relaxing urethral sphincter in 29 (30%), and delayed relaxation of the urethral sphincter in 7 (7%) PwMS. DUA was found in 22 PwMS (23%). All indices demonstrated low to moderate agreement with the diagnosis of DUA and obstruction in this cohort.

**Conclusion:**

Abnormal voiding patterns are common in PwMS, especially sphincter dysfunction. None of the developed indices for obstruction and DUA appear highly relevant in this specific neurogenic population.

## Trial Registration

This study was registered on clinicaltrials.gov: NCT04928716.

## 1 Introduction

Multiple sclerosis (MS) is an autoimmune disorder of the central nervous system characterized by inflammatory demyelination and axonal transection. Lower urinary tract (LUT) dysfunctions are frequent in MS, with a median onset of approximately 8 years after disease initiation [1]. In MS, storage and voiding symptoms are both well described [2]. Urodynamic patterns underlying storage symptoms are clearly defined, but assessment of voiding phase in neurological diseases remains challenging. The International Continence Society (ICS) recommends pressure‐flow studies for evaluating the voiding phase; however, the use of obstruction and contractility indices in neurogenic patients has not been validated [2, 3]. In 2018, the ICS described three types of sphincter dysfunctions: detrusor‐sphincter dyssynergia (DSD) defined as “a detrusor contraction concurrent with an involuntary contraction of the urethral and/or periurethral striated muscle. Occasionally flow may be prevented altogether.”, non‐relaxing urethral sphincter as “a non‐relaxing, obstructing urethral sphincter resulting in reduced urine flow” and delayed relaxation of the urethral sphincter as “an impaired and hindered relaxation of the sphincter during voiding attempt resulting in delay of urine flow” [2]. Neurogenic detrusor underactivity (DUA) is defined as “a contraction of reduced strength and/or duration, resulting in prolonged bladder emptying and/or a failure to achieve complete bladder emptying within a normal time span in the setting of a clinically relevant neurologic disorder” whereas neurogenic acontractile detrusor “is one that cannot be demonstrated to contract during urodynamic studies”. Urodynamic studies in MS have not consistently applied this classification. First, because these definitions are recent, and second, because assessing sphincter relaxation and detrusor contraction during the voiding phase is challenging. In non‐neurogenic patients, the ICS has recently proposed recommendations on pressure‐flow studies, but emphasized the need for further studies to confirm the performance of calculation tools, especially in women [3, 4]. Assessment of sphincter function during pressure‐flow studies is not included in ICS recommendations [3]. Different methods exist to assess the urethral sphincter (electromyography [EMG], urethral pressure, videourodynamics), but each has limitations [5]. Consequently, the physicians conclude from the non‐formalized urodynamic findings whether there is DSD or DUA. In MS, studies have reported 43% of DSD and 12% of impaired contractility [6]. These results should be interpreted cautiously due to variability in assessment methods.

This study aims to describe voiding patterns in patients with MS (PwMS) using standardized assessments and to compare the performance of the available nomograms and indices for evaluating obstruction and bladder contractility.

## 2 Materials and Methods

### 2.1 Study Design

This prospective observational study was conducted in a neuro‐urology department of a French university hospital between 2019 and 2021.

### 2.2 Procedure

All the MS patients consulting for a first urodynamic evaluation without urinary treatment were included. The standardized urodynamic evaluation included uroflowmetry, urethral pressure profile, and two pressure‐flow studies. If no detrusor contractility was observed during the pressure‐flow studies, an ice‐water test was performed to assess detrusor contractility. As a reflection of urethral sphincter activity, EMG of the external anal sphincter (EAS) was recorded during the urodynamic testing using surface electrodes. Urodynamic evaluation was performed in accordance with ICS recommendations.

Demographic data (age, sex), disease course of MS, treatments, Expanded Disability Status Scale (EDSS), urinary symptoms using the Urinary Symptom Profile (USP), and the Neurogenic Bladder Symptom Score (NBSS) were collected. Absence of voiding symptoms was defined with a USP low stream score of 0.

### 2.3 Detrusor Contractility Assessment [3, 7]

Without consensus for assessment of detrusor contractility in patients with neurological disease, the following parameters were reported: (i) the bladder voiding efficiency (BVE) defined as a percentage (BVE = [voided volume/total bladder capacity] × 100); (ii) the bladder contractility index (BCI)—BCI = PdetQmax + 5Qmax—with a strong contractility if BCI > 150, normal contractility if BCI of 100−150 and weak contractility if BCI < 100; (iii) the Watts factor (WF)—WF = ([Pdet + a][vdet + b] − ab)/2π where Pdet is the detrusor pressure, vdet is the velocity of detrusor contraction, and a (25 cmH_2_O) and b (0.6 cm/s) are back‐calculated muscle constants representing the heat of shortening and the rate of energy liberation, respectively.

For women, we used three additional parameters: (i) the PIP1 = PdetQmax + Qmax; (ii) the Valentini−Besson−Nelson (VBN) parameter k (simulating detrusor force); and (iii) a urodynamic cut‐off (Gammie et al.) for DUA with PdetQmax < 20; Qmax < 15, and BVE < 90%.

In the absence of validation of these different indices in neurogenic patients, the conclusion on detrusor contractility resulted from physicians' experience, based on qualitative analysis of urodynamics traces and interpretation of indices. To limit biases, a double reading and interpretation of the urodynamic traces was done. In case of disagreement, all authors analyzed the urodynamic traces to reach a consensus.

### 2.4 Bladder Outlet Obstruction (BOO) and Sphincter Dysfunction Assessment [4, 7]

Without consensus on assessing BOO in patients with neurological disease, the bladder outlet obstruction index (BOOI) was reported: BOOI = PdetQmax − 2Qmax. As thresholds were recently revised, both thresholds of 20 and 40 to define obstruction were presented [7]. For women, we also reported the BOOIf (female BOOI)—BOOIf = PdetQmax − 2.2Qmax—with BOOIf < 0, low probability of obstruction, BOOIf > 5 likely obstructed, and if BOOIf > 18, obstruction almost certain [8].

Sphincter dysfunctions were classified according to the three definitions mentioned above (DSD, non‐relaxing urethral sphincter, and delayed relaxation of the urethral sphincter), using the following parameters, in this preferential order: 1—EMG traces after permission to void when interpretable, 2—shape of the uroflowmetry curve and detrusor pressures—variations in flow rate along with variations in detrusor pressure are suggestive of DSD, and 3—results of the voiding cystourethrography when available. The conclusions on BOO and sphincter dysfunctions were obtained with a double‐blind reading process, as previously described.

### 2.5 Statistical Analysis

Statistical analyses were performed with R Studio software (version 2023.06.2 + 561, R Foundation for Statistical Computing, Vienna, Austria). Means, percentages, and standard deviation (SD) were used to describe the population. The relationships between different contractility and BOO measurement parameters were assessed with Welch's test for quantitative variables and Fisher's test for categorical variables. The diagnostic value of each parameter was evaluated with sensitivity, specificity, positive predictive value (PPV), and negative predictive value (NPV). Agreement between the diagnosis of DUA and BOO according to the used parameters was evaluated using weighted kappa (wkappa). A *p* < 0.05 was considered statistically significant.

### 2.6 Ethics

All the patients provided non‐opposition for the collection and processing of their data. This study was approved by a local ethics committee. All the data were anonymized, and the study was performed in accordance with European regulation no. 2016/679, known as the General Regulation on Data Protection. This study was registered on clinicaltrials.gov: NCT04928716.

## 3 Results

### 3.1 All Patients

Ninety‐seven PwMS were included. The mean age was 48.4 years (SD 10.5), 58 were women (60%), and the mean EDSS was 3.9 (SD 1.8) (Table 1).

**Table 1:** Characteristics of study participants (*N* = 97 patients).

	Mean (SD)/*n* (%)
Age (years)	48.4 ± 10.5
Sex	
– Female	58 (59.8%)
– Male	39 (40.2%)
EDSS	3.9 ± 1.8
Disease course	
– RRMS	68 (70.1%)
– SPMS	14 (14.4%)
– PPMS	15 (15.5%)
DMT	
– None	34 (35.1%)
– Injectables	17 (17.5%)
– Oral therapies	37 (38.1%)
– Monoclonal antibodies	9 (9.3%)
USP	
– USP SUI	1.5 ± 2.3
– USP OAB	7.6 ± 3.9
– USP LS	2.3 ± 2.4
NBSS	
– Incontinence	8.8 ± 6.2
– Storage and voiding	11.1 ± 3.0
– Consequences	2.4 ± 2.8
– Quality of life	2.1 ± 1.3
– Total	24.3 ± 9.5
Urodynamic results of the storage phase	
– DO	54 (55.7%)
– Impaired bladder compliance	1 (1.0%)
– Volume at permission to void	324.8 ± 143.3
VCUG available	59 (60.8%)

Abbreviations: DMT, disease‐modifying treatments; DO, detrusor overactivity; EDSS, expanded disability status scale; LS, low stream; NBSS, neurogenic bladder symptom score; OAB, overactive bladder; PPMS, primary progressive multiple sclerosis; RRMS, relapsing remitting multiple sclerosis; SD, standard deviation; SPMS, secondary progressive multiple sclerosis; SUI, stress urinary incontinence; USP, urinary symptoms profile; VCUG, voiding cystourethrography.

During urodynamic testing, 54 pwMS (56%) had detrusor overactivity (DO). In 5 pwMS (5%), no detrusor contractility could be demonstrated. Twenty‐four (25%) patients had only involuntary detrusor contractions, 39 (40%) had no DO and voluntary detrusor contractions after permission to void, and 29 (30%) had both DO and detrusor contractions after permission to void.

Based on the USP low stream score, 69 (71%) PwMS reported at least one symptom of voiding difficulty. Only 16 patients had normal voiding phases on urodynamics. Abnormal urodynamic findings during the voiding phase were strongly associated with voiding symptoms (19% in PwMS with USP low stream = 0 vs. 96% in those with USP low stream ≥ 1; *p* < 0.001).

DUA or acontractile detrusor were found in 22 PwMS (23%), more frequently in patients without DO during the filling phase (35% vs. 13%; *p* = 0.02). DUA was associated with higher age (52.8 ± 10.1 vs. 47.2 ± 10.2; *p* = 0.03).

Sphincter dysfunction was found in 68 patients (70%), with DSD in 32 (33%) being the most frequent finding, followed by non‐relaxing urethral sphincter in 29 (30%) and delayed relaxation of the urethral sphincter in 7 (7%). DSD occurred only in patients with DO during the filling phase, affecting 74% of this subgroup (*p* < 0.001). The median EDSS score was higher in patients with BOO (median EDSS 4 [2.5−6] vs. 3 [2–4]; *p* = 0.03).

Twelve (12%) PwMS had both contractility impairment and BOO.

### 3.2 Analysis Between Gender Groups

Voiding phase parameters according to gender are described in Table 2. Gender was not significantly associated with a higher risk of BOO (*p* = 0.11) or impaired contractility (*p* = 0.65).

**Table 2:** Evaluation of voiding phase.

	Women mean (SD)/*n* (%)	Men mean (SD)/*n* (%)
Impaired contractility (%)	14 (24.1%)	8 (20.5%)
‐ Acontractile detrusor	4 (6.9%)	1 (2.6%)
‐ Detrusor underactivity	10 (17.2%)	7 (17.9%)
Sphincter dysfunction (%)	39 (67.2%)	29 (74.5%)
‐ DSD	18 (31.0%)	14 (35.9%)
‐ Non‐relaxing urethral sphincter	19 (32.8%)	10 (25.6%)
‐ Delayed relaxation of the urethral sphincter	2 (3.5%)	5 (12.8%)
Bladder outlet obstruction of benign prostatic hyperplasia	—	5 (12.8%)
Post‐void residual (mL)	65 ± 91	75 ± 131
Bladder voiding efficiency (%)	66.2 ± 40.1	61.8 ± 47.6
‐ BVE < 90	31 (53.5%)	
PIP 1	55.5 ± 21.2	—
‐ PIP1 < 30	6 (10.9%)	
BCI	114.6 ± 40.0	106.9 ± 25.1
‐ BCI < 100	20 (34.5%)	14 (35.9%)
WF	8.4 ± 5.1	9.4 ± 2.7
‐ WF < 7	28 (48.3%)	6 (16.7%)
k	0.8 ± 0.6	—
Gammie et al. urodynamic cut‐offs		
DUA = PdetQmax < 20; Qmax < 15, BVE < 90%	4 (6.9%)	—
Mean post‐void residual (mL)	108.9 ± 142.2	94.5 ± 127.7
BOOI	—	38.4 ± 23.2
‐ BOOI > 20		29 (80.6%)
‐ BOOI > 40		17 (43.6%)
BOOIf	8.1 ± 30.0	—
‐ BOOIf > 5	27 (46.6%)	
‐ BOOIf > 18	18 (31.0%)	
Type of detrusor contraction during voiding		
‐ None (acontractile)^a^	4 (6.9%)	—
‐ Detrusor overactivity	14 (24.1%)	10 (25.6%)
‐ Contractility after permission to void	25 (43.1%)	14 (35.9%)
‐ Both detrusor overactivity and contractility after permission to void	15 (25.9%)	14 (35.9%)

*Note:* Number of patients: 58 women (3 women with no possibility to void during pressure flow study) and 39 men (3 men with no possibility to void).

Abbreviations: BCI, bladder contractility index; BOOI, bladder outlet obstruction index; BOOIf, female bladder outlet obstruction index; BVE, bladder voiding efficiency during uroflowmetry before catheterization; DSD, detrusor‐sphincter dyssynergia; DUA, detrusor underactivity; PIP1, projected isometric pressure 1; SD, standard deviation; WF, Watts factor.

a Four women obtained voiding without detrusor contractility (3 with abdominal straining and 1 with perineal relaxation).

Data for the different parameters used in the diagnosis of DUA are presented in Table 3. Post‐void residual (PVR) was significantly higher in men with DUA (252 ± 170 vs. 54 ± 74 mL; *p* < 0.001), whereas it was not significant in women (148 ± 110 vs. 82 ± 115 mL; *p* = 0.07).

**Table 3:** Comparison of presence of DUA and contractility index.

	Women			Men		
	DUA (*n* = 14) Mean ± SD or *N* (%)	Non‐DUA (*n* = 41) Mean ± SD or *N* (%)	*p*	DUA (*n* = 6) Mean ± SD or *N* (%)	Non‐DUA (*n* = 30) Mean ± SD or *N* (%)	*p*
PIP1	36.4 ± 11.1	62.0 ± 20.0	< 0.001			
‐ PIP1 < 30^a^	6 (42.8%)	0	< 0.001			
BCI	78.3 ± 25.1	127.0 ± 36.6	< 0.001	82.8 ± 23.2	112 ± 22.8	0.008
‐ BCI < 100^a^	11 (78.6%)	9 (22.0%)	< 0.001	5 (83.3%)	9 (30.0%)	0.014
Bladder voiding efficiency (%)	45.1 ± 43.5	72.1 ± 37.5	0.037	56.8 ± 48.3	63.2 ± 48.1	0.75
‐ BVE < 90^a^	11 (78.6%)	20 (48.8%)	0.07			
WF	4.6 ± 1.5	9.7 ± 5.2	< 0.001	7.0 ± 2.6	9.9 ± 2.5	0.012
‐ WF < 7^a^	14 (100.0%)	14 (34.1%)	< 0.001	3 (50.0%)	3 (10%)	0.016
k	0.37 ± 0.24	0.95 ± 0.57	< 0.001			
Gammie et al. urodynamic cut‐offs^a^	4 (28.6%)	0	< 0.001			
Mean post‐void residual (mL)	148 ± 110	82 ± 115	0.07	252 ± 170	54 ± 74	< 0.001

*Note:* Number of patients: 55 women (3 women of 58 with no possibility to void during pressure flow study) and 36 men (3 men of 39 with no possibility to void).

Abbreviations: BCI, bladder contractility index; BOOI, bladder outlet obstruction index; BOOIf, female bladder outlet obstruction index; BVE, bladder voiding efficiency during uroflowmetry before catheterization; DUA, detrusor underactivity; Gammie et al. urodynamic cut‐offs: DUA, PdetQmax < 20; Qmax < 15, BVE < 90%; PIP1, projected isometric pressure 1; SD, standard deviation; WF, Watts factor.

^a^ Fisher test.

Conversely, PVR was higher in women with BOO (134 ± 154 vs. 57 ± 99 mL; *p* = 0.03) while no significant difference was observed in men (94 ± 117 vs. 99 ± 180 mL; *p* = 0.95). The etiology of obstruction was not associated with higher PVR. Parameters for BOO diagnosis are presented in Tables 4 and 5. Five men (5%) presented benign prostatic hyperplasia, two of whom also had associated sphincter dysfunction (mixed obstruction).

**Table 4:** Comparison of the presence of sphincter dysfunction and obstruction index in women.

	DSD (*n* = 15)	Non‐relaxing urethral (*n* = 19)	Delayed relaxation of the urethral sphincter (*n* = 2)	No sphincter dysfunction (*n* = 19)	*p*
	Mean ± SD or *N* (%)	Mean ± SD or *N* (%)	Mean ± SD or *N* (%)	Mean ± SD or *N* (%)	
BOOIf	31.9 ± 29.1	8.2 ± 27.8	6.3 ± 19.3	−10.4 ± 20.1	< 0.001
‐ BOOIf > 5^a^	12 (80%)	10 (53%)	1 (50%)	4 (21%)	0.004
‐ BOOIf > 18^a^	10 (67%)	3 (32%)	1 (50%)	1 (5%)	< 0.001
Mean post‐void residual (mL)	170 + /−191	109 ± 113	48 ± 59	57 ± 99	0.10

*Note:* Number of patients: 55 women (3 women of 58 with no possibility to void during pressure flow study).

Abbreviations: BOOI, bladder outlet obstruction index; BOOIf, female bladder outlet obstruction index; DSD, detrusor‐sphincter dyssynergia; SD, standard deviation.

^a^ Fisher test.

**Table 5:** Comparison of the presence of bladder outlet obstruction and obstruction index in men.

	DSD (*n* = 12)	Non‐relaxing urethral (*n* = 8)	Delayed relaxation of the urethral sphincter (*n* = 5)	No bladder outlet obstruction (*n* = 6)	Obstruction by BPH (*n* = 3)	Mixt obstruction (*n* = 2)	*p*
	Mean ± SD or *N* (%)	Mean ± SD or *N* (%)	Mean ± SD or *N* (%)	Mean ± SD or *N* (%)	Mean ± SD or *N* (%)	Mean ± SD or *N* (%)	
BOOI	40.9 ± 17.6	38.7 ± 23.4	42.8 ± 6.6	15.1 ± 16.2	59.4 ± 40.9	50.0 ± 41.9	0.09
‐ BOOI > 20	11 (92%)	6 (75%)	5 (100%)	2 (33%)	3 (100%)	2 (100%)	0.06
‐ BOOI > 40^a^	7 (58%)	3 (37%)	4 (80%)	0	2 (66.7%)	1 (50.0%)	0.08
Mean post‐void residual (mL)	99 ± 112	124 ± 172	24 ± 18	99 ± 180	75 ± 22	123 ± 14	0.85

*Note:* Number of patients: 36 men (3 men of 39 with no possibility to void).

Abbreviations: BOOI, bladder outlet obstruction index; BPH, benign prostatic hyperplasia; DSD, detrusor‐sphincter dyssynergia; Mixt obstruction, bladder outlet obstruction by both BPH and sphincter dysfunction; SD, standard deviation.

^a^ Chi‐square test.

The diagnostic performances of each parameter for DUA and BOO are presented in Tables 6 and 7, respectively. Across genders, all indices showed only low to moderate agreement with the diagnosis of DUA and obstruction.

**Table 6:** Diagnostic value and reliability of thresholds of contractility parameters in people with multiple sclerosis.

	PPV	NPV	Sensitivity	Specificity	Cohen's kappa (*p* value)
Women					
PIP1 < 30	100%	83.7%	42.7%	100%	0.53 (*p* < 0.001)
BCI < 100	55.0%	91.4%	78.6%	78.0%	0.50 (*p* < 0.001)
BVE < 90	35.5%	87.5%	78.6%	51.2%	0.21 (*p* = 0.052)
WF < 7	50.0%	100.0%	100.0%	65.9%	0.50 (*p* < 0.001)
Gammie urodynamic cut‐offs	100.0%	80.4%	28.6%	100.0%	0.37 (*p* < 0.001)
Men					
BCI < 100	35.7%	95.5%	83.3%	70.0%	0.35 (*p* = 0.014)
WF < 7	50.0%	90.0%	50.0%	90.0%	0.40 (*p* = 0.016)

*Note:* Number of patients: 55 women (3 of 58 women with no possibility to void during pressure flow study) and 36 men (3 men of 39 with no possibility to void).

Abbreviations: BCI, bladder contractility index; BVE, bladder voiding efficiency during uroflowmetry before catheterization; DUA, detrusor underactivity; Gammie urodynamic cut‐offs: DUA, PdetQmax < 20; Qmax < 15, BVE < 90%; NPV, negative predictive value; PIP1, projected isovolumetric pressure 1; PPV, positive predictive value; WF, Watts factor.

**Table 7:** Diagnostic value and reliability of thresholds of bladder outlet obstruction parameters in people with multiple sclerosis.

	PPV	NPV	Sensitivity	Specificity	Cohen's kappa (*p* value)
Women					
BOOIf > 5	85.2%	53.6%	63.9%	78.9%	0.39 (*p* = 0.003)
BOOIf > 18	94.4%	48.6%	47.2%	94.7%	0.34 (*p* = 0.002)
Men					
BOOI > 20	93.1%	57.1%	90.0%	66.7%	0.53 (*p* = 0.002)
BOOI > 40	100.0%	31.6%	56.7%	100.0%	0.30 (*p* = 0.02)

*Note:* Number of patients: 55 women (3 of 58 women with no possibility to void during pressure flow study) and 36 men (3 men of 39 with no possibility to void).

Abbreviations: BOOI, bladder outlet obstruction index; BOOIf, female bladder outlet obstruction index; NPV, negative predictive value; PPV, positive predictive value.

## 4 Discussion

In our study, we found a high rate of voiding dysfunction in PwMS who were not receiving any treatment for bladder disorders, with 83.5% showing abnormal urodynamic voiding patterns. The majority also reported subjective voiding difficulties. In most cases (70%), voiding dysfunction was related to urethral sphincter dysfunction, mainly DSD (32%) and non‐relaxing urethral sphincter (29%).

The rate of voiding dysfunction in PwMS in our study is one of the highest in the literature. However, our population does not seem clinically different than other cohorts previously published based on mean age, EDSS, and NBSS score [9, 10]. One important point is the lack of systematic assessment of voiding difficulties in this population. Most of the questionnaires used focus on storage symptoms (NBSS, OABq, OABSS…), whereas only a few papers reported scores of voiding symptoms. Li et al. described a smaller population of 40 PwMS (mean age 50), with a mean USP low stream of 1.61, and a mean USP OAB of 6.4 [11], whereas in a cohort of 202 patients without any medication for bladder symptoms, a median USP OAB of 7, and a median USP low stream of 2 was reported [12]. Moreover, using the IPSS score, Ziadeh et al. described a median IPSS‐V of 8, with 88% of patients reporting mixed symptoms of storage and voiding phase, with a median EDSS of 3 [13]. Voiding difficulties are usually less frequently reported by patients than storage symptoms, as it impacts quality of life on a lower level. Thus, they could easily be missed during clinics, as patients do not report them spontaneously.

Indication of urodynamics in MS is based on individual assessment. If urodynamics is not mandatory before introducing a treatment for OAB symptoms, the presence of voiding difficulties can lead to recommending urodynamics, and this could partly contribute to the high rate of voiding difficulties reported in our cohort, in which urodynamics was a criterion of inclusion.

On urodynamics, we found sphincter dysfunction in 70% of cases, with 32% of DSD. This finding is consistent with a systematic review of 1524 PwMS across 13 studies, which reported DSD in 43% of cases [6]. However, the reported prevalence of DSD varied widely, ranging from 16% in Tadayyon et al. to 71.0% in Ukkonen et al. [14, 15]. Several factors may explain these discrepancies. First, the definition of DSD varies across studies, and many did not specify how they defined DSD. Second, the assessment methods of DSD varied substantially between studies. For instance, EMG has been used in several studies, sometimes with needle electrodes, in different muscles (external urethral sphincter [16, 17, 18], EAS [14], bulbocavernosus muscles [17]), sometimes with surface electrodes [19, 20]. Some studies used fluoroscopy to define DSD, either alone or in combination with EMG recording [18, 19, 20, 21]. Other studies provided no information about the method used to define DSD [22, 23, 24]. Moreover, these methods have inherent limitations [5]. EMG accuracy can be impacted by the placement of the electrodes, the type of electrode used, and pain induced by the needle in the urethral sphincter. In video‐urodynamics and pressure‐flow studies, the presence of the catheter can induce a urethral reflex. The ability to void as usual may be disturbed by the catheter or the position required to record images, leading to an overestimation of voiding dysfunction. Despite being described as the gold standard, videourodynamic is not widely available, costly, and time‐consuming. The use of urethral pressure to explore DSD has also been described, but it seems poorly reliable compared to fluoroscopy [25].

It has been suggested that DSD can be diagnosed with only pressure‐flow studies, by the characteristic hydrodynamic pattern that flow occurs when the pressure drops in the relevant clinical context [26]. This is appropriate in patients with spinal cord injury (suprasacral spinal cord dysfunction), but in MS, presentation is heterogeneous, as demyelination occurs in different sites, with different degrees of severity. The degree of brain and spinal atrophy may also play a significant role in disabilities and bladder symptoms. DSD is commonly associated with spinal lesions, and its prevalence depends on the severity of the disease in the studied population. In our cohort, the mean EDSS score was 3.9 ± 1.9, and obstruction was associated with higher EDSS, supporting the probable spinal involvement in sphincter dysfunction. However, the presence of spinal plaque is not sufficient to conclude on a spinal reflex responsible for bladder dysfunction as described in spinal cord injury, and some labeled DSD could in fact be dysfunctional voiding related to a learnt behavior in response to OAB symptoms.

Other types of sphincter dysfunctions have been poorly described. Barbalias et al. reported 10% of incomplete sphincter relaxation [18]. The pathophysiology of incomplete sphincter relaxation is unclear, but it could be related more to a sphincteric dysfunction, leading to the maintenance of a guarding reflex and thus reduced detrusor contraction during voiding. On the contrary, one hypothesis in dysfunctional voiding is a primary bladder dysfunction leading to a learned abnormal voiding behavior. Thus, a higher rate of DO and higher detrusor pressure during the voiding phase are reported in dysfunctional voiding compared to poor relaxation of the urethral sphincter in non‐neurological women [27]. A confusion between these types of dysfunctions and DSD across studies cannot be excluded, especially when the method to assess DSD is unclear.

DUA was not common in our cohort, with only 23% of patients, consistent with prior studies reporting impaired contractility in 12% of cases [6]. We found a modest association with older age. This may reflect age‐related decline in bladder contractility [28]. Interestingly, bladder emptying was not impaired in women with DUA, probably due to the ability to compensate with straining, in the absence of BOO, facilitated by the short length of the urethra and the low bladder outlet resistance in women. On the contrary, we found a higher PVR in women with obstruction, whereas it was not significant in men. This could be related to a lack of power, as only five male individuals did not have obstruction.

One main limitation in our study is the definition of DUA and obstruction. Indeed, the validated indices have not been used in neurogenic populations. Obstruction indices have been developed for anatomical obstruction. In neurogenic bladder, especially in MS, where patients are relatively young, obstruction is mainly related to a dynamic process due to sphincter dysfunction. Thus, obstruction was defined using a combination of arguments, including voiding cystography, pressure‐flow study, and surface electrodes EMG. The low Cohen's kappa coefficients reflect the poor reliability of these indices to define BOO in this population. Similarly, contractility indices demonstrated poor agreement in men and intermediate in women. Another difficulty is the interpretation of voiding when DO occurs at the same time. A recent study in women with neurogenic bladder found increased BOOIf in patients with DO‐related voiding compared with voluntary voiding [29].

In the last recommendations on pressure‐flow studies, the interpretation of terminal DO‐voiding is appropriate only if micturition reflects the patient's usual behavior [7].

Whether the type of sphincter dysfunction changes the therapeutic management remains unclear. The gold standard for managing neurogenic voiding dysfunction remains intermittent self‐catheterization. However, in the absence of risk factors and DSD, pelvic floor muscle relaxation, alpha blockers, and maybe intrasphincteric injections of botulinum toxin could be discussed, despite the lack of evidence.

This study has some limitations. Interpretation of DUA and BOO remained partly subjective in the absence of formal definitions. Other medications than bladder treatment may impair contractility, but only a small number of patients had an anticholinergic drug score over 3, minimizing the potential iatrogenic effect. MRI data were not available; thus, it was not possible to correlate DSD with the presence of spinal lesions. Finally, whether pressure‐flow studies represent the usual bladder behavior is unclear. The representativeness with daily life must always be questioned as technical conditions can impact voiding, as well as the sensory stimulation by the urethral catheter, the temperature of the water filling, the anxiety related to the test, etc. Further studies using catheter‐free bladder pressure monitoring would help to differentiate the abnormal voiding patterns in patients with neurogenic bladder [30].

## 5 Conclusion

Abnormal voiding patterns are common in PwMS, with obstruction being the most frequent (70%). Detrusor‐sphincter dyssynergia was found in 32% of patients, always in association with detrusor overactivity, but other sphincter dysfunctions, such as poor urethral sphincter relaxation or delayed relaxation, are also common. Impaired contractility is less common, concerning 23% of individuals. None of the currently available indices for obstruction and bladder contractility appear relevant in this population.

## Author Contributions

**Camille Chesnel:** conceptualization (equal), writing – original draft (lead), formal analysis (lead), investigation (equal). **Nicolas Turmel:** conceptualization (equal), writing – review and editing (equal), investigation (equal). **Maelys Teng:** conceptualization (equal), writing – review and editing (equal), investigation (equal). **Emilie Blouet:** conceptualization (equal), investigation (equal), project administration (lead). **Gérard Amarenco:** conceptualization (equal), writing – review and editing (equal), supervision (lead). **Claire Hentzen:** conceptualization (equal), writing – original draft (supporting), writing – review and editing (equal), investigation (equal).

## Funding

The authors have nothing to report.

## Ethics Statement

All the patients provided non‐opposition for the collection and processing of their data. This study was approved by a local ethics committee.

## Conflicts of Interest

The authors declare no conflicts of interest.

## Data Availability

The data that support the findings of this study are available from the corresponding author upon reasonable request.
